# Molecular Dynamics Simulations of Double-Stranded DNA in an Explicit Solvent Model with the Zero-Dipole Summation Method

**DOI:** 10.1371/journal.pone.0076606

**Published:** 2013-10-04

**Authors:** Takamasa Arakawa, Narutoshi Kamiya, Haruki Nakamura, Ikuo Fukuda

**Affiliations:** Institute for Protein Research, Osaka University, Suita, Osaka, Japan; University of Bologna & Italian Institute of Technology, Italy

## Abstract

Molecular dynamics (MD) simulations of a double-stranded DNA with explicit water and small ions were performed with the zero-dipole summation (ZD) method, which was recently developed as one of the non-Ewald methods. Double-stranded DNA is highly charged and polar, with phosphate groups in its backbone and their counterions, and thus precise treatment for the long-range electrostatic interactions is always required to maintain the stable and native double-stranded form. A simple truncation method deforms it profoundly. On the contrary, the ZD method, which considers the neutralities of charges and dipoles in a truncated subset, well reproduced the electrostatic energies of the DNA system calculated by the Ewald method. The MD simulations using the ZD method provided a stable DNA system, with similar structures and dynamic properties to those produced by the conventional Particle mesh Ewald method.

## Introduction

The static and dynamic structural features of nucleic acids, DNA and RNA, and their complexes with proteins are essential for their biochemical functions and the regulation of gene expression during transcription, replication, and translation [1]. Since the solution structures of nucleic acids are generally more flexible than those of globular proteins, their structural plasticity should always be considered. Thus, although many X-ray crystal structures of nucleic acids have been solved, their dynamic structures with the structural ensemble should also be extensively analyzed. For that purpose, molecular dynamics (MD) simulations are frequently utilized for effective investigations [2]–[5].

In MD simulations of nucleic acids, intra- and inter-molecular electrostatic interactions play fundamental roles because of their highly charged and polar features, in addition to their long-range nature [6]. The charges located at the backbone phosphate groups make the DNA and RNA polymers negatively charged, and thus positive counter-ions are distributed closely along the phosphate backbones for neutralization. In fact, the conformation of double-stranded DNA is stabilized by the “condensation” of the counterions and the associated Deby-Hückel type screening process, where the phosphate negative charges are substantially neutralized by the counterions [7], [8].

A straight cutoff truncation of the electrostatic interactions is frequently applied in MD simulations, but this resulted in many artifacts, since the long distance effects were simply neglected [9], [10]. In particular, an MD simulation with such a cutoff procedure for double-stranded DNA systems profoundly deforms the initial double-stranded forms [11].

An alternative approach with lattice sum techniques, such as the Ewald method [12] and the Particle mesh Ewald (PME) method [13], [14], has been recommended for MD simulations of DNA systems, assuming the periodic boundary condition. Here, the long-range electrostatic interactions are not ignored, and many physicochemical properties of the periodic systems are reproduced.

Although the lattice sum approach is the most popular standard technique, its applications to intrinsically non-periodic systems clearly deviate from reality [15]. Recently, the artifacts produced by the simple cutoff method have been significantly reduced by several new approaches, referred to as the non-Ewald methods, which were reviewed in detail elsewhere [15]. Among them, we have developed the zero-dipole summation (ZD) method [16]–[18], which takes into account the neutralities of charges (zero-monopole) and dipoles (zero-dipole) in a truncated subset. The ZD method can be viewed as an extension of the other non-Ewald method developed by Wolf et al. [19], [20], and it provided more accurate electrostatic energies for a liquid NaCl system [16], a pure TIP3P water system [17], and a membrane protein system [18].

One of the advantages of the ZD method is its rapid computation. The durations of single and parallel calculations using the ZD and PME methods were evaluated in our previous studies for a pure TIP3P water system [17] and a membrane protein system [18]. The real space part of the PME method with a shorter cutoff length, in principle, requires less computational time than that of the ZD summation method. However, we can utilize the effectiveness of the ZD method without the complementary error function, and the differences between the results of the distinct cutoff values in the practical cutoff region in both systems tend to be small, as the number of processors increases [17], [18]. Because the ZD method is free from evaluation of the reciprocal Fourier part required in the lattice sum method, an *O*(*N*) scheme is implied for a large system by computing only local interactions. Thus, we expect an effective scalability in the current method. In fact, we have obtained positive results for several large biomolecular systems [21]. Another advantage of the ZD method comes from the fact that the ZD method is irrelevant to the boundary conditions. Thus, it does not need to obey the exact periodic boundary condition in application to an intrinsically non-periodic system.

Here, we applied the ZD method to a double-stranded DNA dodecamer, d(5′-CGCGAATTCGCG-3′)_2_, with a precisely determined X-ray crystal structure including explicit water molecules and small ions. We confirmed that the electrostatic energies of the current DNA system were very well reproduced as compared to those calculated by the Ewald method, and that the MD simulations using the ZD method provided a stable DNA system, with similar structures and dynamic properties to those produced by the PME method.

## Methods

### The algorithm of the ZD method

A brief description of the algorithm of the ZD method are provided here, instead of the detailed derivation and features of the method [16], [17]. The total Coulombic electrostatic energy, *E_total_*, can be described by



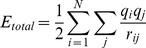
(1)




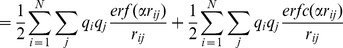
(2)


where *q_i_* is the atomic charge of the *i*’th atom and 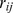 is the distance between *i*’th atom and *j*’th atom. The manner of the summation with respect to *j* depends on the boundary conditions. The error function and the complementary error function are designated as *erf* and *erfc*, respectively, with a damping factor *α* (≥0).

In the Ewald method with the periodic boundary conditions, the first term of Eq. 2 can be evaluated via the Fourier space term and the self-energy term. While, the non-Ewald methods [15], such as the Wolf method [19], [20] and the ZD method [16]-[18], utilize the fact that the first term of Eq. 2 for a small *α* can be approximated by only a self-energy term 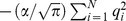
and especially vanishes at all for *α* = 0. Regarding the second term of Eq. 2, a truncation with a short cutoff can be employed for a relatively large *α*, as in the Ewald approach. Instead, in order to provide an efficient estimate of the second term even for a small *α*, the theoretical frame of the ZD method assumes that the interaction contribution is counted in a certain neutralized subset *M_i_*, which consists of certain particles inside the cutoff sphere of an individual *i*’th atom. The subset *M_i_*, including the *i*’th atom, is characterized such that the total sums of the charges and dipole moments are both zero in *M_i_*, and any atoms not belonging to *M_i_* but inside the cutoff sphere are located close to the surface. Note that the creation of subset *M_i_* is not actually required in practical applications, because *M_i_* is introduced conceptually to express the theoretical viewpoint. That is, via mathematical considerations with respect to the approximation of the excess energy [16], we can convert the summation of the original potential function *erfc*(*αr*)/*r* over *M_i_* into an ordinary pair-wise summation of a suitably reconstructed potential function inside the cutoff sphere (See Eqs. 10 and 41 in Ref. 16). This results in an approximation of Eq. 2 as



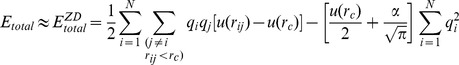
(3)




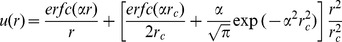
(4)


Here, in contrast to the lattice sum method, the assumption of the total charge neutrality in the MD cell is not necessarily required. For a general system with molecules having covalent bonds, the relevant modifications to Eq. 3 are required [17].

### Double-stranded DNA dodecamer

**Figure 1:**
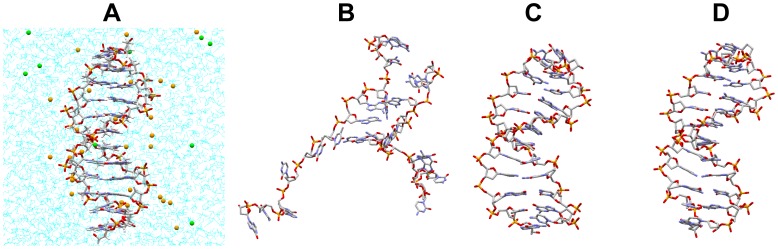
The double-stranded DNA dodecamer system. (A) The initial DNA structure (PDB 1bna) with the explicit TIP3P water molecules (cyan), Na^+^ ions (orange) and Cl^−^ ions (green). The atom colors of DNA are the CPK colors. (B) A snapshot at 50 ps by the RESA method with *r_c_* = 18 Å. (C) A snapshot at 10 ns by the ZD method, with *r_c_* = 12 Å and *α* = 0.0. (D) A snapshot at 10 ns by the PME method, with *r_c_* = 12 Å and *α* = 0.35 Å^−1^. Hydrogen atoms are not shown in (B) to (D).

The atomic coordinates of a double-stranded DNA dodecamer, d(5′-CGCGAATTCGCG-3′)_2_, were obtained from the Protein Data Bank (PDB) [22] (PDBID 1bna [23]). This DNA model was embedded in a box with dimensions of 80 Å x 60 Å x 60 Å. In total, 9,048 explicit TIP3P water molecules [24] and 39 Na^+^ and 17 Cl^−^ ions were added in the box, for a physiologically neutral environment. The total number of atoms in the box was 27,958 (Figure 1 (A)). The initial steepest descent energy minimization (811 steps) was followed by conjugated gradient minimizations (982 steps) with positional restraints on the solute, using a force constant of 10 kcal/(mol Å^2^). The system was then equilibrated for 1 ns by adopting Berendsen’s NPT algorithm [25] with temperature and pressure coupling at 300 K and 1 atm, respectively, with the periodic boundary condition using the PME method [14] with the real part cutoff of 12 Å and a damping factor *α* = 0.35 Å^−1^ for electrostatic interactions, and with a time step of 0.5 fs. The cutoff distance of the van der Waals interactions was 12 Å. After this equilibration run, the NVT production run at 300 K was performed by either the PME or ZD method with the cell size (85.150×55.088×58.327 Å^3^) and a time step of 1.0 fs. The SHAKE algorithm was applied to the system. The charges of the DNA atoms and the force field were originated from AMBER parm99bsc0 [26]. The MD simulations by the PME method and the energy calculations by the Ewald method were performed by using cosgene/myPresto [27].

In addition, to assess the accuracy of the ZD method quantitatively, the energies were computed by both the Ewald and ZD methods for 1,000 snapshot structures taken every 1 ps, from the 1 ns MD trajectory, which was produced using the PME method for generating physically plausible phase-space points.

## Results and Discussion

**Figure 2:**
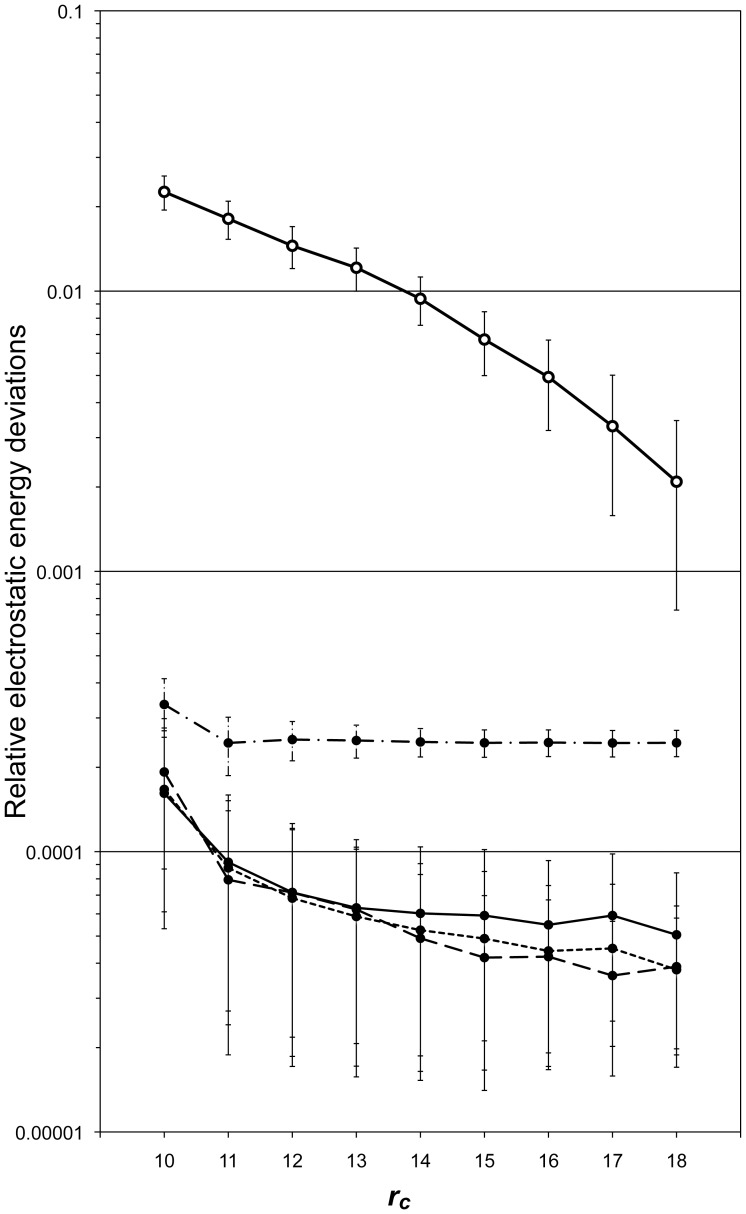
Relative electrostatic energy deviations by the ZD and RESA methods with the cutoff distance *r_c_*, averaged for 1,000 sampled structures produced by MD with the PME method, compared with those calculated by the Ewald method (Eq. 5). The thin solid line, dotted line, dashed line, and dash-dotted line with filled circles are the results from the ZD method with damping factor *α* values  =  0.0, 0.06, 0.10, and 0.14 (Å^−1^), respectively. The thick solid line with open circles represents the results from the RESA method. The error bars are the standard deviations for 1,000 samples.

First, the total electrostatic energies of the double-stranded DNA system were computed by the ZD method, *E^ZD^*, depending on the cutoff distance, for the 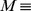
1,000 snapshot structures, 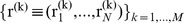. They were compared to those calculated by the Ewald method, 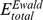. The resultant average relative deviations are defined by



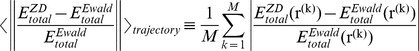
(5)


and are shown in Figure 2. The atom-based cutoff procedure was used in the ZD method.

For comparison with the simple and straight truncation method, the total energies by the residue-based cutoff method of the bare Coulombic function (the RESA method), 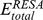, were also calculated by the following equation:
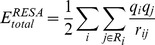
(6)


In Eq. 6, *R_i_* is a group of interacting atoms for the *i*’th atom, where all of the interactions in a residue are counted, if at least one atom in the residue is inside the cutoff sphere, centered on the *i*’th atom. For 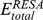, the relative deviations from 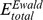 were also calculated in the same manner as in Eq. 5, and are shown in Figure 2.

It is clear that the deviation of 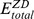 from 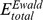 is very small when *α* is equal to or less than 0.1 Å^−1^, even at a short cutoff distance. In fact, the relative deviation was only about 0.007% at *r_c_* = 12 Å when *α* = 0.0, and it decreased to about 0.005% at *r_c_* = 18 Å. In contrast, 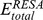 deviated much more than 1.0% from 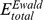 at the 12 Å cutoff distance, and it decreased to about 0.2% at the 18 Å cutoff distance, which is still much larger than the deviation observed for the ZD method with *α* smaller than 0.1 Å^−1^.

In the cases of the pure water system [17] and the membrane protein system [18], the ZD method with *α* = 0.0 always provided the best approximations at *r_c_* values larger than 10 Å. However, in the current DNA system, the ZD method with *α* = 0.06 Å^−1^ or 0.1 Å^−1^ sometimes gave better approximations than those with *α* = 0.0, although the differences are within the standard deviations for 1,000 samples. On the contrary, the total energy calculated by the ZD method with *α* = 0.14 Å^−1^ always deviated from the Ewald method much more than those with the smaller *α* values. This feature is similar to those observed in our previous reports [17], [18], and its origin can be identified as a consequence of the approximation of the first term of Eq. 2. The current study revealed that the ZD method with small *α* values also works effectively for a highly charged inhomogeneous DNA system at short *r_c_*, thus allowing us to use a very small or even zero damping factor without the annoying choice of the parameter value.

Since the system is not homogeneous, in order to dissect the contributions from each molecule and nucleotide, we investigated the electrostatic energy contributed from the *i*’th atom, 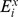, which is defined below considering the interaction energy between the *i*’th and *j*’th atoms, 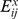:
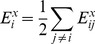
(7)

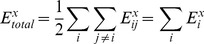
(8)


Here, *x* designates the method used to calculate the electrostatic energies: *Ewald*, *ZD*, and *RESA*, respectively.

**Figure 3:**
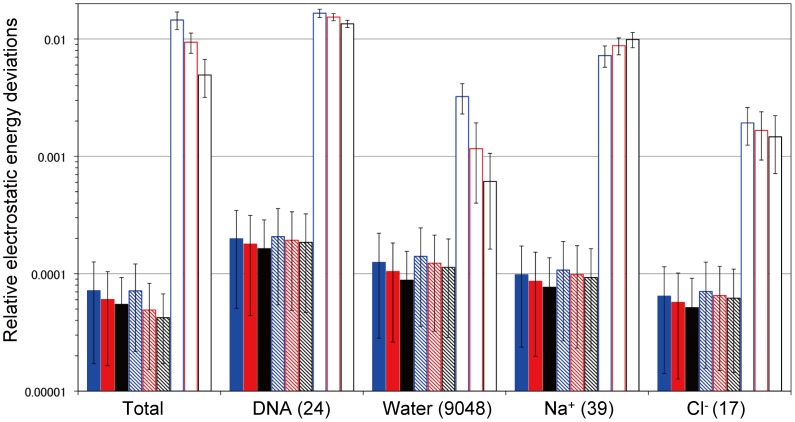
Relative individual contributions to electrostatic energy deviations by the ZD and RESA methods from the Ewald electrostatic energy, averaged for 1,000 sample structures produced by MD with the PME method (Eq. 9). Solid blue, red, and black bars are the contributions by the ZD method with the cutoff distance *r_c_* values  =  12, 14, and 16 (Å), respectively, and the damping factor *α* = 0.0. Hatched blue, red, and black bars are those with *r_c_* = 12, 14, and 16 (Å), respectively, with *α* = 0.1 (Å^−1^). Open blue, red, and black bars are those obtained by the RESA method with *r_c_* = 12, 14, and 16 (Å), respectively. The numbers in parentheses are the numbers of nucleotides in DNA, the numbers of water molecules, and the numbers of Na^+^ and Cl^−^ ions. The error bars are the standard deviations for 1,000 samples.



The average contributions from individual molecules in the energy deviations from the Ewald electrostatic energy were determined by the following Eq. 9, and they are shown in Figure 3.

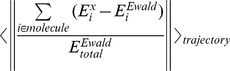
(9)In Eq. 9, *x* is *ZD* with *α* = 0 and 0.1 (Å^−1^), and *RESA* is used for comparison. Here, the *molecule* is the double-stranded DNA, all 9,048 waters, 39 Na^+^, and 17 Cl^−^. The total deviation in Eq. 5 is also shown.

The energy accuracies for DNA, Na^+^ and Cl^−^ ions were all about hundred times better with the ZD method than the RESA method. On the contrary, the accuracies for TIP3P water were about 10 times better with the ZD method than the RESA method, as in the membrane protein system [18].

**Figure 4:**
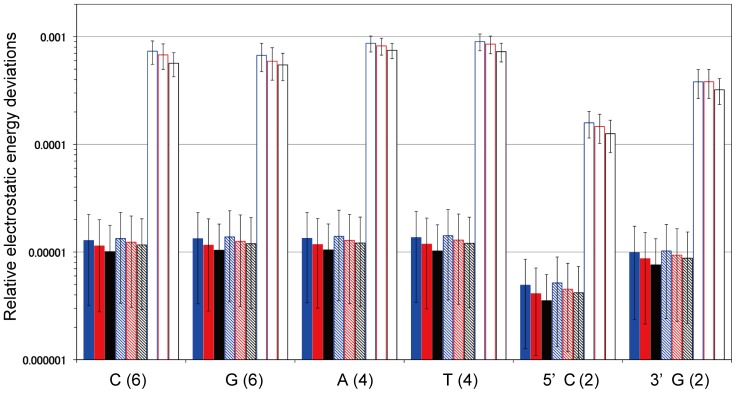
Relative contributions of each nucleotide to the electrostatic energy deviations, determined by the ZD and RESA methods from the Ewald electrostatic energy averaged for 1,000 sample structures produced by MD with the PME method (Eq. 10). The parameters for the individual bars are the same as those in Figure 3. The numbers in parentheses are the numbers of nucleotide types. The error bars are the standard deviations for 1,000 samples.



The contribution of each nucleotide in the energy deviation from the Ewald electrostatic energy was computed, to understand which nucleotide type contributed to the total energy deviation. The following values in Eq. 10 were calculated for the individual nucleotides of the current DNA model with 24 nucleotides: for the 4 different nucleotides, and the 5' and 3' terminal nucleotides, and they are shown in Figure 4.

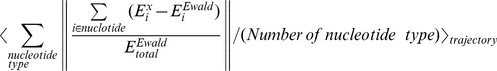
(10)Here, *x* is *ZD* and *RESA*. The number of each nucleotide type is described in parentheses after each nucleotide type in Figure 4. The accuracies with the ZD method were twenty- to seventy-times better than those with the RESA method. The deviations with the ZD method using *α* = 0 and *α*  = 0.1 (Å^−1^) were similar, and they were smaller for larger *r_c_* in every residue. From the feature that the 5'-terminal nucleotides lack the phosphate groups with large negative charge atmosphere, the deviations for the 5'-terminal nucleotides in both the ZD and RESA methods were about half of those for the other nucleotides, respectively.

One concern may arise about the assumption applied by the ZD method in the current inhomogeneous DNA system for the neutrality of the monopole and dipole moments, which is satisfied in the large ensemble of homogeneous systems, such as the NaCl liquid [16] and pure water systems [17]. For an inhomogeneous protein system, it was confirmed that the monopole and dipole moments in a rather restricted local region are small enough to be well approximated by the ZD method [18]. In the current DNA system, although the 5'-terminal nucleotides lack the negatively charged phosphate groups, the anti-parallel double-stranded structure does not generate any large dipole moment. In addition, the counterion screening should provide neutral conditions, even around the phosphate groups. As well, it is also suggested that significant portion of phosphate charge neutralization is performed by water molecules hydrating the DNA [28]. Therefore, for the inhomogeneous DNA system, the monopole and dipole moments can be small enough to be well approximated by the ZD method.

As described in the Introduction section, a simple truncation of the electrostatic interactions generally deforms DNA structures. In fact, as shown in Figure 1(B), the double-stranded DNA in the current system was completely deformed after only a 50 ps MD simulation by the RESA method with *r_c_* = 18 Å. On the contrary, even after a 10 ns MD simulation by the ZD method with *r_c_* = 12 Å and *α* = 0.0, the structure of the DNA (Figure 1 (C)) was maintained in a similar manner to that by the PME method, with *r_c_* = 12 Å and *α* = 0.35 Å^−1^ (Figure 1 (D)).

**Figure 5:**
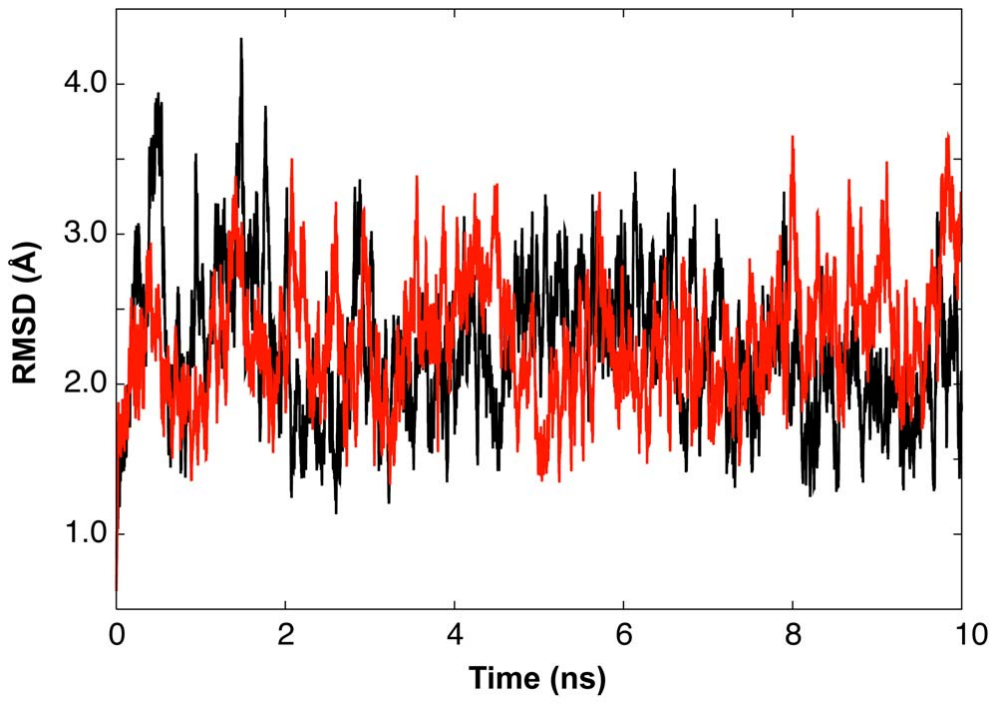
The trajectories of the rmsd values of the backbone heavy atoms of the double-stranded dodecamer DNA from those atoms of the initial crystal structure, along 10 ns MD simulations with the PME method (black line, *r_c_* = 12 Å and *α* = 0.35 Å^−1^) and with the ZD method (red line, *r_c_* = 12Å and *α* = 0.0). Both methods started from the same initial structure.

The 10 ns MD trajectories of the root-mean square deviations (rmsds) from the initial crystal structure, obtained by the PME method with *r_c_* = 12 Å and *α* = 0.35 Å^−1^ and the ZD method with *r_c_* = 12 Å and *α* = 0.0, are shown in Figure 5 by black and red lines, respectively. After relatively large fluctuations at initial 1–2 ns, the motions indicate the equilibrium, and the rmsds obtained by the ZD method were similar to those generated by the PME method. This shows that the stabilities of the systems in the MD simulations by the ZD method and the PME method were comparable.

The absolute values of the rmsds depend on the initial configuration state we prepared, and this would explain the relatively large value of the rmsds. Relevant to this observation, in order to analyze the dynamic properties of the DNA in more detail, we examined the root-mean-square fluctuations (rmsf) of the backbone atoms, averaged along the MD trajectories. For this analysis, the double-stranded DNA snapshot structures with A- and B-chains were first obtained at every 1 ps, and they were superimposed on the initial crystal structure. Then, the average value of each backbone heavy atom position 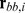 was calculated as 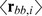. The rmsf value for the backbone atoms of the *m*’th nucleotide was thus calculated as:
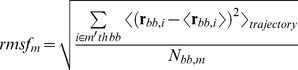
(11)


**Figure 6:**
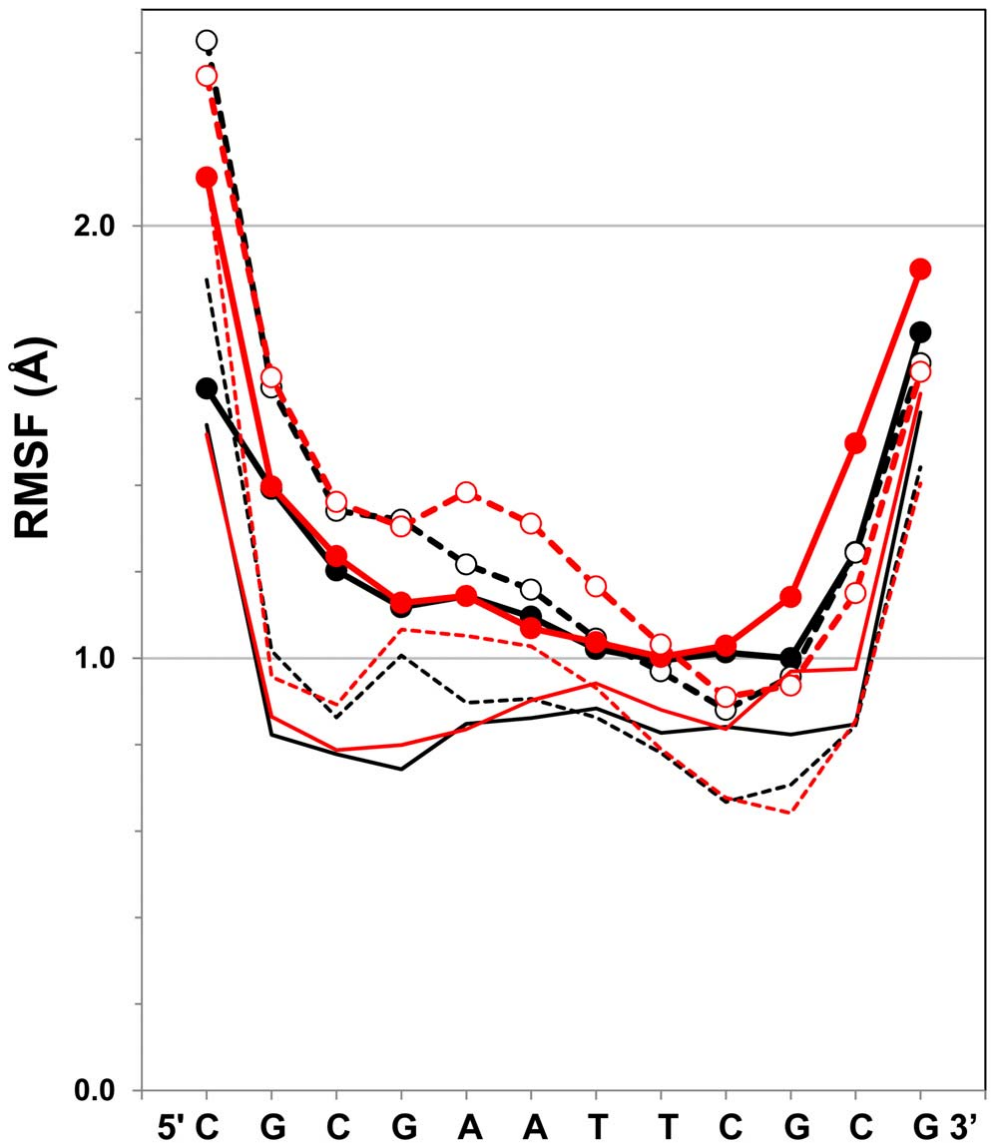
The rmsf (root-mean-square fluctuation) values of the backbone heavy atoms (thick solid lines with filled circles for A-chain, and thick dotted lines with open circles for B-chain), and that of the base heavy atoms (thin solid lines for A-chain, and thin dotted lines for B-chain), averaged along the last 7 ns of the total 10 ns MD trajectories shown in Figure 5 by the PME method (black lines, *r_c_* = 12 Å and *α* = 0.35 Å^−1^) and the ZD method (red lines, *r_c_* = 12 Å and *α* = 0.0). The abbreviations are used for deoxyribonucleotides: C, cytosine; G, guanine; A, adenine; T, thymine.

Here, *N_b,m_* is the number of atoms located in the backbone of the *m*’th nucleotide. The rmsf values for all of the nucleotides in both the A- and B-chains are plotted by thick lines in Figure 6 for the last 7 ns of the total 10 ns MD trajectories by the PME and ZD methods, which are shown in Figure 5. In the same manner, the rmsf values for the base heavy atoms were also calculated, and are shown by thin lines in Figure 6.

The nucleotide flexibility reflected by the rmsf values depended on the relative position in the double-stranded DNA chains. The rmsf values of the terminal nucleotides were generally large for the backbone atoms, including the sugar atoms, and the base atoms. In particular, the 5’-terminal cytosine backbones were quite flexible, for which the pyrimidine base rings frequently moved during the MD simulations. In MD simulations for similar short double-stranded DNA models with explicit water molecules, the terminal flexibility was also found [29], which were emphasized for cytidines [30]. Although the base atoms at both the termini were fairly flexible, the base atoms within the 2^nd^ to 11^th^ nucleotides were stable enough to give very small rmsfs less than 1 Å, due to the hydrogen bonds between the base-pairs and the stacking interactions between the adjacent bases. Relatively small fluctuations of the base against the backbone were found in an intensive study of MD simulations on an oligonucleotide in the B-DNA form [30].

Since the rmsf values obtained by both the PME and ZD methods closely coincide in Figure 6, the flexible features observed in the PME method are well reproduced in detail by the ZD method. In fact, the Pearson correlation coefficients of the rmsf values between the PME method and the ZD method were 0.944 and 0.977 for the backbone heavy atoms, and the base heavy atoms, respectively. The overall correlation coefficient was 0.969 for all of the heavy atoms of DNA.

The cross-correlations at the individual nucleotides for the fluctuations of the backbone heavy atoms, and those of the base heavy atoms, averaged along the trajectories, were also examined by monitoring the following cross-correlation matrix element *C_ij_*:



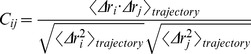
(12). 

**Figure 7:**
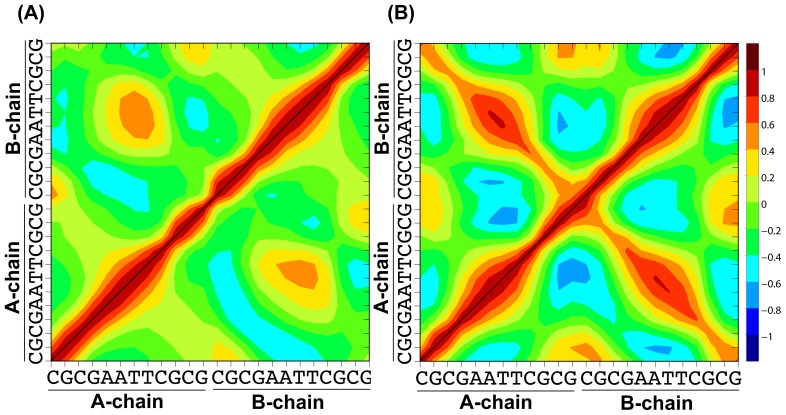
The cross-correlation matrix elements in the fluctuations of (A) the DNA backbone heavy atoms, and (B) the base heavy atoms, averaged along the trajectories of the last 7 ns of the total 10 ns MD simulations. The cross correlation values from 1.0 to –1.0 are colored red (positive) to blue (negative). The lower triangles show the values obtained by the PME method (*r_c_* = 12 Å and *α* = 0.35 Å^−1^), and the upper triangles show the correlations provided by the ZD method (*r_c_* = 12 Å and *α* = 0.0), respectively. The A- and B-chains are indicated with the nucleotide abbreviated names.

Here 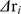 is the displacement from the mean position of the backbone and base atoms of the *i*’th nucleotide. A positive cross correlation indicates in-phase displacement, while a negative correlation is out-of-phase. In Figure 7, the averaged matrix elements are plotted during the last 7 ns of the 10 ns MD simulations shown in Figure 5. The upper triangle shows the correlations obtained by the ZD method, and the lower triangle shows those generated by the PME method. The local dynamic correlations for the ZD method and the PME method coincide very well even at the terminal nucleotides, which are very flexible. The Pearson correlation coefficients of each *C_ij_* element between the PME method and the ZD method were 0.976 and 0.983 for the backbone heavy atoms and for the base heavy atoms, respectively. The overall correlation coefficient was 0.982 for all of the heavy atoms, including the backbones and bases. Typical positive correlations are observed in Figure 7, in particular at the base-pairs with stable hydrogen bonds and the adjacent bases with stacking interactions. They correspond to the distinctive concerted fluctuations, and these characteristic behaviors were captured by both methods.

The results presented in Figures 5–7 indicate that the ZD method provides the dynamic properties of an inhomogeneous, highly charged molecular system as correctly as the MD simulation with the conventional PME method.

The PME method devises the fast calculation protocol for the Ewald summation by utilizing the fast Fourier transform, and has been a standard in the electrostatic evaluation, in particular at an early stage [31]. However, in the area of MD simulation on nucleic acid, other protocols have been frequently applied [32], [33], followed by the pioneering works [34]–[36]. In fact, the atom-based shifted force cutoff method has often been used [37]–[44], for which the efficiency has been dictated through the systematic and complete study by Norberg and Nilsson [30].

Although we admit these successes on this method, our concern is its physical basis for the successful simulations. One possible interpretation is due to the screening effect, but we still do not have complete answer why and how it makes a difference in various systems. Consistency between the handling of the electrostatic interaction and the bonding interaction is also not perfectly explained. While we have the idea that the shifted-force potential should be assessed on only the 1–5 (1–4) non-bonded pairs [45], we recognize the idea that they should be on all the pairs along with the removal of the pure coulomb (not shifted) form of the all bonding 1–2, 1–3 pairs. The latter handling conforms to the zero-charge Wolf [19], [20] and the zero-dipole methods [16], [17]. In fact, the revised no-damping Wolf method [46] is equivalent to the shifted force method in view of the pairwise formula, whose potential function can be expressed as 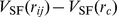 with 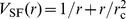, having force 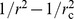. Nevertheless ambiguities in an actual conduct of such methods are already discussed in Ref. [15], [20].

Another effective cutoff method is the reaction field (RF) method [47]–[49], which has a certain physical basis, in contrast. It takes into account the interactions between each molecule and the environment outside the cutoff sphere, or cavity, of the molecule. The region outside the cavity is viewed as a uniform, homogeneous dielectric continuum polarized via reacting with the molecules inside the cavity. In spite of its success, only a few applications of the RF method to double-stranded DNA system were attempted. This may be because the assumption of the homogeneity repels the application of this method to highly heterogeneous media such as those including the DNA. Against such low motivation, the RF method was utilized by Kastenholz et al. to investigate the transition between the B and Z conformations of DNA with explicit-solvent [50] and by Nina and Simonson for a RNA hairpin in solution, obtaining several positive results [51]. In particular, Ni and Baumketner [52] applied the RF method successfully into a DNA tetradecamer with explicit solvent and revealed the importance of the atom-based cutoff mode. The pros and cons of the RF method are discussed in Ref. [15].

The ZD method utilizes the local feature for the electrostatic neutrality, and its physical basis is discussed in detail in the previous studies [15]–[17]. In the current study we have revealed at first that the ZD method is successfully applied to the very inhomogeneous and highly charged DNA system in explicit solution. Detailed investigations of the energy accuracy, such as done in the current study, have not been pursued in the above methods for the application to DNA, to the best of our knowledge. These indicate that the ZD method should be an alternation of the electrostatic calculation method in DNA systems. This study conducts the positive and successful MD simulation of a DNA system, followed by crystal and liquid NaCl systems [16], a pure TIP3P water system [17], and a membrane protein with lipid bilayer molecules, explicit water molecules, and ions [18].

Finally, as a practical point, we discuss the computation performance of the ZD method for the current DNA system. The computation times required in single and parallel calculations using the ZD method were evaluated, and they are shown in Table 1, in comparison with those of the PME method. For this purpose, we focus on the real space part of the PME method in the same manner for a homogeneous water system and a heterogeneous GPCR system discussed previously [17],[18], because the evaluation of the reciprocal Fourier part highly depends on the parallel algorithm and architecture. In general, the real space part of the PME method with a shorter cutoff length requires a shorter computational time than that of the ZD summation method. However, as shown in Table 1, the differences between the results of the distinct *r*_c_ values in the practical cutoff region tend to be small, as the number of processors increases. As well as the accuracy, in view of the speed performance, the ZD method with *α* = 0 has an advantage because of the elimination of the complementary error function. The full PME method needs to add the calculation of the reciprocal Fourier part. Thus, the computation time required by the ZD method with a long *r*_c_ value could become shorter than that by the PME method including the reciprocal space part.

**Table 1:** Execution time (sec) for 1 step MD simulation of the DNA system by the ZD method and by the real space part of the PME (RPME) method.

Method: *r*_c_, *α*	*n* = 1	*n* = 8	*n* = 16	*n* = 32
ZD: 10, 0	1.3643	0.2659	0.1890	0.1613
ZD: 12, 0	1.6518	0.3044	0.2051	0.1796
ZD: 12, 0.1	1.9662	0.3443	0.2241	0.1870
ZD: 14, 0	2.0404	0.3544	0.2326	0.1877
RPME*: 10, 0.35	1.5401	0.2881	0.1964	0.1819
RPME*: 12, 0.35	1.9559	0.3359	0.2244	0.1943

Cutoff length *r*_c_ (Å) and damping factor *α* (Å^−1^) are indicated; *n* is the number of processors. *: Execution time with a single processor (*n* = 1) for the reciprocal part of the PME method was 0.14 sec.

## Conclusions

The molecular simulation of DNA system via the atom-based shifted-force cutoff method without using the lattice sum method under the periodic boundary condition for evaluation of the electrostatic interactions was unexpectedly successful [53]. However, this success was also achieved by both the RF method [52] and the current ZD method. These studies suggest that non-Ewald cutoff-based methods can be applied to highly charged and polar systems if we employ them with certain proper methodologies. In particular, we clarified this fact in an application of the ZD method to an explicit solvent DNA model, by investigations of the rmsd and rmsf of the heavy atoms, the cross-correlations in fluctuations of the backbone heavy atoms, and the accuracy of the energy.

In general, cutoff-based methods are attractive in view of their simplicity, which enables fast MD calculations. An investigation focusing on similarities or the relationship among these methods is important, because it may lead us to capture common physics framework underlying these otherwise theoretically different-looking methods. This expectation is supported by the current study using the ZD method. In fact, a physical assumption in the ZD method regarding the existence of the neutralized subset in a highly charged and polar DNA system was not trivial until a realistic application was performed. Although the current study provides a positive view for the validity of such an assumption, it could be the case that the success is due to other physical logic. Such reconsiderations may also be required for both the force shift method, whose physical base is still unclear, and the RF method, whose success under the homogeneity assumption is not yet completely understood. It is crucial to pursuit a general logic and provide a new physical view for explaining the success of non-Ewald methods and facilitate their theoretical advances.
